# Modeling the Multidimensional Predictors of Multisite Musculoskeletal Pain Across Adulthood—A Generalized Estimating Equations Approach

**DOI:** 10.3389/fpubh.2021.709778

**Published:** 2021-08-11

**Authors:** Ville-Heikki Ahlholm, Viljami Rönkkö, Leena Ala-Mursula, Jaro Karppinen, Petteri Oura

**Affiliations:** ^1^Center for Life Course Health Research, Faculty of Medicine, University of Oulu, Oulu, Finland; ^2^Medical Research Center Oulu, University of Oulu and Oulu University Hospital, Oulu, Finland; ^3^Finnish Institute of Occupational Health, Oulu, Finland; ^4^Department of Forensic Medicine, University of Helsinki, Helsinki, Finland

**Keywords:** musculoskeletal pain, lifestyle, socioeconomic, occupational exposure, psychological factors, epidemiology, cohort studies

## Abstract

**Background:** Multisite pain is commonly chronic and often lacks its initial role as a potential tissue damage signal. Chronic pain among working-age individuals is a risk for disability and imposes a major burden on health care systems and society. As effective treatments for chronic pain are largely lacking, better identification of the factors associated with pain over working years is needed.

**Methods:** Members of the Northern Finland Birth Cohort 1966 participated in data collection at the ages of 31 (*n* = 4,028) and 46 (*n* = 3,429). Using these two time points, we performed a multivariable analysis of the association of socioeconomic, occupational, psychological and lifestyle factors (i.e., low education, living alone, low household income, unemployment, occupational physical exposures [hard physical labor, leaning forward, back twisting, constant moving, lifting loads of ≥ 1 kg], physical inactivity, regular smoking, regular drinking, overweight, and psychiatric symptoms) with the number of musculoskeletal pain sites (i.e., upper extremity, lower extremity, lower back, and the neck-shoulder region; totalling 0–4 pain sites). The data were analyzed using generalized estimating equations.

**Results:** At the age of 31, multisite pain was reported by 72.5% of men and 78.6% of women. At the age of 46, the prevalence of multisite pain was 75.7% among men and 82.7% among women. Among men, the number of pain sites was positively associated with age (rate ratio 1.05, 95% confidence interval 1.01–1.08), low household income (1.05, 1.01–1.08), unemployment (1.13, 1.06–1.19), any occupational exposure (1.17, 1.12–1.22), regular smoking (1.06, 1.02–1.11), and psychiatric symptoms (1.21, 1.17–1.26). Among women, the number of pain sites was positively associated with age (1.06, 1.04–1.10), unemployment (1.10, 1.05–1.15), any occupational exposure (1.10, 1.06–1.13), regular smoking (1.06, 1.02–1.10), overweight (1.08, 1.05–1.11), and psychiatric symptoms (1.19, 1.15–1.22); living alone was negatively associated with the number of pain sites (0.95, 0.91–0.99).

**Conclusion:** Of the studied predictors, psychiatric symptoms, occupational physical exposures and unemployment were most strongly associated with multisite pain among both sexes. The results of this study deepen the understanding of the underlying factors of and comorbidities behind multisite pain, and help develop pain relief and rehabilitation strategies for working-age individuals with multisite pain.

## Background

Pain is an unpleasant experience, traditionally perceived as a signal of actual or potential tissue damage (1). It is always experienced subjectively and regulated by biological and psychosocial factors (2). Multisite or widespread pain is defined as pain occurring in more than one anatomical site, and it is common among adults globally (1). Multisite pain is typically chronic, and it is no longer associated with tissue damage (3). Chronic pain among working-age individuals imposes a major burden on health care systems and society (2). As there is a lack of effective treatments and therapeutic modalities for chronic pain, the factors associated with pain status over working years need to be more accurately identified.

The study of multisite pain and its underlying factors has gained scientific interest internationally. Several studies suggest that multisite pain is more common among females than males (4–7) and among old than young individuals (4). Multisite pain has also been associated with psychological factors and psychiatric symptoms such as depression, anxiety, fatigue and low levels of self-care (8–14). One study found that the number of pain sites was higher among individuals with socioeconomic hardships, e.g., those divorced or separated, undergoing rehabilitation and on a disability pension (5). Individuals may also develop multisite symptoms because of their physical workload (15). As for health-related lifestyle factors, evidence has accumulated that multisite pain is associated with smoking, physical inactivity and obesity (5, 6, 11, 16).

Most previous evidence is limited by its cross-sectional setting, although pain status and its determinants may fluctuate across adulthood. In addition, only a few studies have been able to simultaneously model a wide range of underlying factors including socioeconomic, occupational, psychological and lifestyle dimensions. It is important to account for all these dimensions together in a comprehensive dataset in order to evaluate their interrelations and reveal independent effects on an individual's pain status across adulthood. Finally, prospective studies with large population-based samples are essential for reaching high general representativeness. Studies focusing on working-age individuals should be of particular importance from the perspective of economic impact and timely rehabilitation.

In this study, we aimed to evaluate the association between multidimensional risk factors and the number of musculoskeletal pain sites at two timepoints in adulthood. As previous studies have revealed several underlying factors of multisite pain, we aimed to perform a comprehensive multivariable analysis, in an attempt to identify individual predictors of pain status from the pool of socioeconomic, occupational, psychological and lifestyle dimensions. With our data, we were also able to compare the identified predictors in terms of effect size. In accordance with previous studies, we hypothesized that the number of pain sites would be associated with older age, indicators of lower socioeconomic status, the presence of occupational exposures, unhealthier lifestyle, obesity, and psychiatric symptoms.

## Methods

### Study Sample

**Figure 1:**
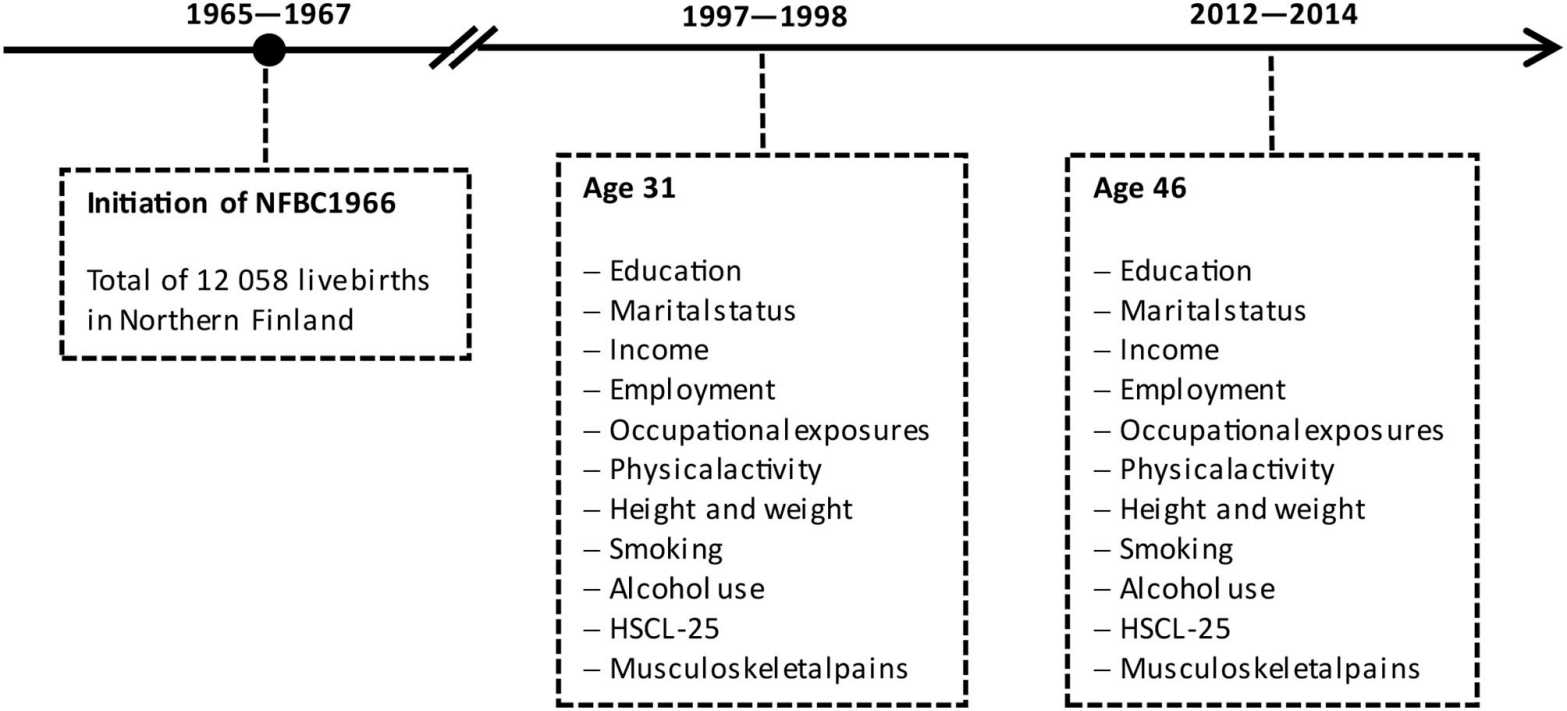
Flow chart of the study. HSCL-25, Hopkins Symptom Checklist-25; NFBC1966, Nothern Finland Birth Cohort 1966.

This study was based on the Northern Finland Birth Cohort 1966 (NFBC1966) population (17), which initially comprised 12,058 live births in 1965–1967 in Northern Finland. In 1997–1998, when the cohort members were 31 years old, they were prompted to respond to a questionnaire on socioeconomic, occupational, psychological and lifestyle factors (*n* = 4,028 respondents). In 2012–2014 when the cohort members were 46 years old, they received a new questionnaire enquiring about the same variables as before (*n* = 3,429 respondents). At both time points, the questionnaires included the same questions on education, marital status, household income, employment, occupational physical exposures, physical activity, smoking status, alcohol use, psychiatric symptoms, and musculoskeletal pains. In addition, the cohort members' height and weight were measured at both times, as part of clinical examinations. All observations were included, i.e., individuals were not excluded if they had data from only 31 or 46 years. The outline of the study is illustrated in Figure 1.

### Assessment of Socioeconomic, Occupational, Psychological, and Lifestyle Factors

Age was calculated on the basis of birth dates. However, as the sample was a 1966 birth cohort and thus coeval, the age variable was dichotomous (31 and 46 years, i.e., first and second time points, respectively).

Basic education was reported on the basis of the following alternatives: (1) Uncompleted compulsory school, (2) Compulsory school, (3) Completion of matriculation examination. Additional education was reported on the basis of the following alternatives: (1) None, (2) Occupational course, (3) Vocational school, (4) Other lower-level institute/academy/college, (5) Polytechnic, (6) University, (7) Other, (8) Not yet completed. Low education was defined as having only compulsory school (18).

Marital status was selected from the following alternatives: (1) Married, (2) Cohabiting, (3) In a registered partnership, (4) Unmarried, (5) Divorced, (6) Divorced from a registered partnership, (7) Widow, (8) Widow from a registered partnership. Individuals who were not married, in a registered partnership or cohabiting, were considered to live alone.

Household income was elicited by asking: “Please report the total income of your household last year (before taxes).” The respondents were also asked to report the number of family members in their household. Household income (per household member) less than the participants' median value at each time point was defined as low household income (19).

Employment status was selected from the following alternatives: (1) Permanent full-time employee, (2) Permanent part-time employee, (3) Temporary full-time employee, (4) Temporary part-time employee, (5) Full-time self-employed or entrepreneur, (6) Part-time self-employed or entrepreneur, (7) Full-time student, (8) Part-time student, (9) Unemployed for <6 months, (10) Unemployed for 6–12 months, (11) Unemployed for > 2 months, (12) Employed/educated through labor market support, (13) Laid off or reduced working hours, (14) Maternity/paternity leave or parental leave, (15) Retired, (16) Caring for my own household, (17) Other. Individuals who were not working or studying full-time or part-time, self-employed or entrepreneurs, employed/educated by labor market support, or on parental leave, were considered unemployed (20).

The presence of several occupational physical exposures (“hard physical labor that is strenuous for the whole body,” “leaning forward,” “having to twist back,” “constant moving or walking from one place to another,” “lifting loads of > 1 kg”) were each elicited separately with the following response alternatives: (1) Never or very rarely, (2) Rarely, (3) Occasionally, (4) Often, (5) Very often. Individuals who reported encountering any of the enquired exposures at least often were considered to have an occupational exposure (21). We also analyzed all exposures separately; in this analysis each exposure formed its own variable, and the response was considered positive if the respective exposure was encountered at least often.

Leisure-time physical activity was elicited by the following question: “How often do you engage in brisk exercise (getting out of breath and sweating)? (1) Once a month or less, (2) 2–3 times a month, (3) Once a week, (4) 2–3 times a week, (5) 4–6 times a week, (6) Daily.” Individuals who reported exercising less than once a week were defined as inactive (20).

Smoking habits were elicited by the following question: “Do you currently smoke? (1) 7 days a week, (2) 5–6 days a week, (3) 2–4 days a week, (4) Once a week, (5) Occasionally, (6) Not at all.” Alcohol consumption was reported by answering the following questions: “Do you consume alcohol, even occasionally (yes/no)?”; “How often do you consume alcohol (beer, cider and long-drinks; wine; strong alcohol—each enquired separately)? (1) Never, (2) Once a year or less, (3) 2 times a year, (4) 3–4 times a year, (5) Once in 2 months, (6) Once a month, (7) 2–3 times a month, (8) Once a week, (9) Several times a week, (10) Daily.” Individuals who reported smoking or drinking more often than once a week were considered regular smokers and drinkers, respectively. While there are no universal definitions for regular smoking (22) or drinking (23), the present cut-offs were selected as they were considered to capture both daily users and also those non-daily users who frequently expose themselves to tobacco or alcohol.

At the clinical examinations, a research nurse systematically measured the height and weight of each participant using calibrated standard scales (i.e., stadiometers for height and bathroom scales for weight), with the participant barefoot and in their underwear. At the 46-year follow-up, height was measured twice, and the mean of two measurements was used as the final height. Body mass index (BMI) was calculated as weight (kg) divided by height (m) squared, and overweight was defined as BMI ≥ 25 kg/m^2^ (24).

Symptoms of anxiety and depression were screened using the validated Hopkins Symptom Checklist-25 (HSCL-25) (25, 26) total mean score. Individuals who scored ≥ 1.55 were defined as having clinically relevant psychiatric symptoms (27, 28).

### Assessment of Musculoskeletal Pains

**Figure 2:**
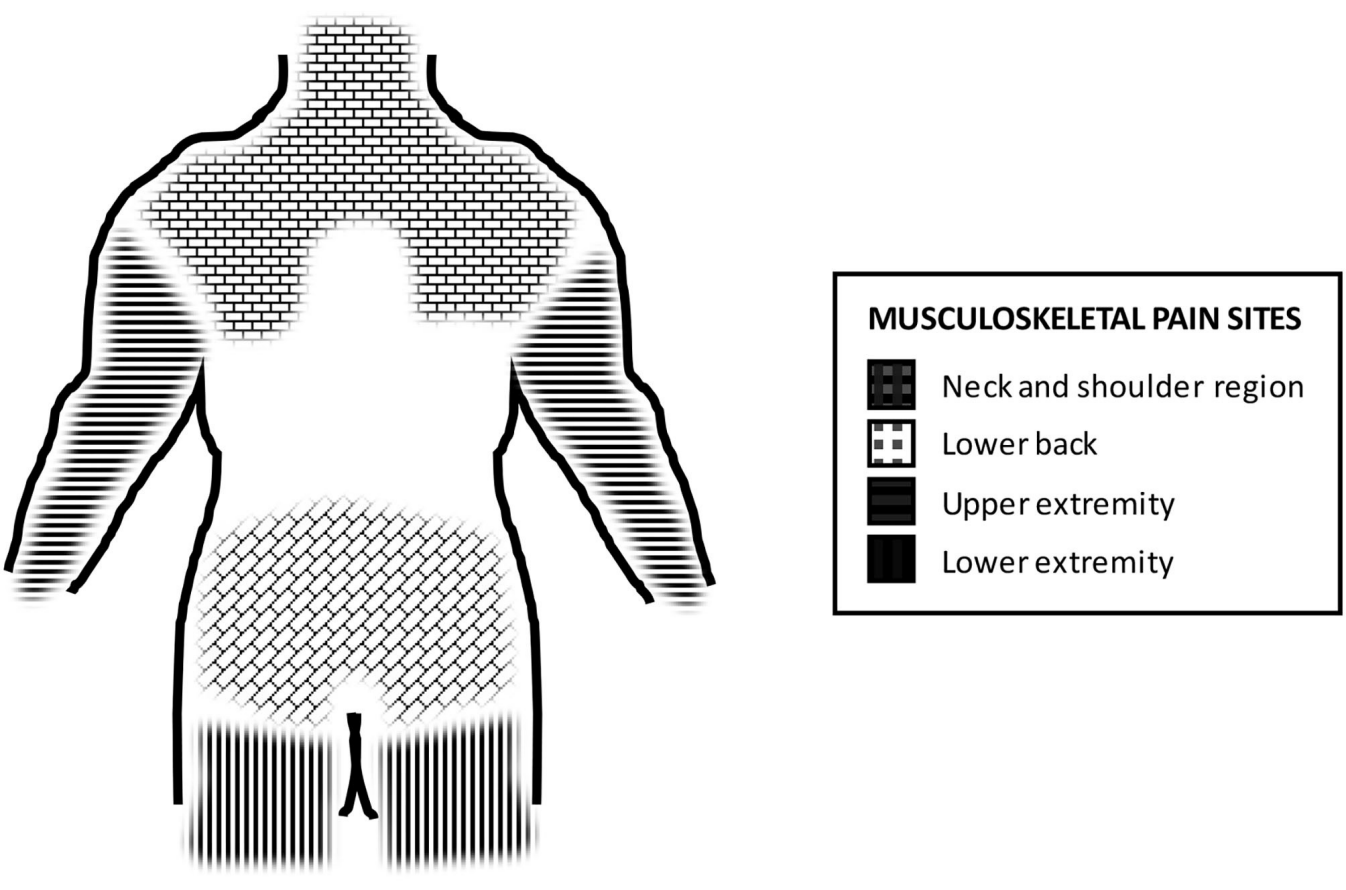
Schematic illustration of the four major musculoskeletal pain sites.

The presence of musculoskeletal pain during the previous year was elicited separately for four anatomical areas: the neck-shoulder region, lower back, upper extremity, and lower extremity (yes/no for each; illustration in Figure 2). The areas were further defined to the respondents by means of a verbal description and/or a visual demonstration (Figure 2). Based on the responses, we constructed the number of musculoskeletal pain sites (total of 0–4 pain sites) variable which acted as the outcome variable in the analyses. Individuals with at least two pain sites were considered to have multisite pain (29).

### Statistical Analysis

Statistical analysis was performed using SPSS version 26 (IBM, Armonk, NY, USA). The threshold for statistical significance was set at *P* = 0.05. As all variables were categorical, their distributions were presented using frequencies and percentages. To evaluate the associations between the predictors (i.e., socioeconomic, occupational, psychological and lifestyle variables) and the outcome (i.e., number of pain sites), we used a generalized estimating equations (GEE) approach. GEE is a regression-based method which is particularly suitable for the analysis of longitudinal or otherwise correlated data, as it accounts for all time points and is able to correct for correlations between variables by introducing a working correlation matrix (30, 31). We selected the negative binomial model with log link and the “exchangeable” working correlation matrix, as it provided the best fit for the data according to the Quasi-likelihood under Independence Model Criterion (QIC) and the Corrected Quasi-likelihood under Independence Model Criterion (QICC).

First, we evaluated the univariate associations between each predictor and the outcome. Then, as our main analysis, we constructed full multivariable models which included all the predictors. Finally, we ran a sub-analysis, which specified the independent roles of each occupational exposure relative to the outcome. All variables except for sex were treated as time varying. As we expected to encounter clear sex differences in the predictors of pain status, we stratified all models by sex. All observations were included, i.e., individuals were not excluded if they had only responded at 31 or 46 years, under the assumption of data missing at random. Exponentiated regression coefficients (i.e., rate ratios, RRs), 95% Wald confidence intervals (CIs), and *P*-values were recorded from the GEE output. RRs represent the relationship between a predictor variable and the outcome over the follow-up.

### Ethical Approval

The study followed the Declaration of Helsinki principles and was approved by the Ethical Committee of the Northern Ostrobothnia Hospital District together with the Scientific Committee of the Northern Finland Birth Cohorts. The study was based on voluntary participation and the cohort members gave their written informed consent to use their data in scientific studies at both time points. The data were processed anonymously.

## Results

Our analysis was based on a total of 3,615 observations of men (*n* = 2,026 and *n* = 1,589 at the ages of 31 and 46, respectively) and 3,842 observations of women (*n* = 2,002 and *n* = 1,840 at the ages of 31 and 46, respectively). Table 1 presents the detailed characteristics of the sample. By the age of 46, 21.7% of men and 21.5% of women had not completed a secondary or tertiary level education. At the age of 31, 48.3% of men and 30.3% of women were overweight or obese, and at the age of 46, these percentages were 68.8 and 51.4%, respectively. At the ages of 31 and 46, multisite pain (i.e., pain in two or more sites) was reported by 72.5 and 75.7% of men and by 78.6 and 82.7% of women, respectively.

**Table 1:** Characteristics of the study population.

**Characteristic**	**Men**					**Women**				
	**Age 31**			**Age 46**		**Age 31**			**Age 46**	
	**%**	***(n)***		**%**	***(n)***	**%**	***(n)***		**%**	***(n)***
Number of individuals		2,026			1,589		2,002			1,840
Low education level^a^	29.3	(594)		21.7	(345)	31.8	(636)		21.5	(395)
Living alone^b^	25.5	(516)		14.9	(236)	23.7	(474)		13.1	(241)
Low household income^c^	46.1	(933)		47.4	(753)	50.7	(1,015)		49.1	(904)
Unemployment^d^	12.5	(254)		11.4	(181)	17.0	(341)		11.4	(209)
Any occupational exposure^e^	58.9	(1,194)		46.8	(743)	60.6	(1,213)		51.5	(948)
Hard physical labor	26.0	(526)		18.5	(294)	16.4	(329)		14.9	(275)
Leaning forward	27.9	(565)		24.6	(391)	35.5	(710)		33.4	(615)
Back twisting	24.4	(495)		21.3	(339)	27.9	(559)		26.6	(489)
Constant moving	42.6	(863)		31.3	(498)	43.2	(865)		35.0	(644)
Lifting loads of ≥ 1 kg	39.9	(809)		29.4	(467)	29.5	(591)		25.1	(462)
Physical inactivity^f^	34.6	(702)		29.9	(475)	32.9	(658)		23.4	(430)
Regular smoking^g^	33.2	(673)		18.4	(293)	23.8	(477)		15.5	(285)
Regular drinking^h^	22.0	(446)		33.7	(535)	9.2	(185)		19.9	(366)
Overweight^i^	48.3	(979)		68.8	(1,094)	30.3	(606)		51.4	(945)
Psychiatric symptoms^j^	13.7	(278)		16.5	(262)	22.6	(453)		21.3	(392)
Number of pain sites^k^										
0	9.8	(199)		7.8	(124)	5.4	(109)		3.8	(70)
1	17.7	(358)		16.5	(262)	16.0	(320)		13.5	(248)
2	23.3	(472)		25.4	(404)	25.3	(507)		23.2	(427)
3	26.2	(531)		26.1	(415)	27.4	(548)		28.5	(524)
4	23.0	(466)		24.2	(384)	25.9	(518)		31.0	(571)
Multisite pain^l^	72.5	(1,469)		75.7	(1,203)	78.6	(1,573)		82.7	(1,522)

a *Primary education only*.

b *Not married, in a registered partnership, or cohabiting*.

c *Total household income divided by the number of household members; low indicates less than the participants' median value*.

d *Not working full time or part time, excluding students, entrepreneurs, those on a parental leave, and those employed by labor market support.*

e *At least one of the listed exposures encountered often at work.*

f *Physically active during leisure time less than once a week.*

g *Smoking regularly more than once a week.*

h *Drinking regularly more than once a week.*

i *Body mass index ≥ 25 kg/m^2^.*

j *Symptoms of depression and anxiety according to Hopkins Symptom Checklist-25 total mean score of ≥ 1.55.*

k *Of the following: neck and shoulder region, lower back, upper extremities, lower extremities.*

l *I.e., at least two pain sites*.

Table 2 presents the results from the GEE analyses. In the full multivariable model of men, the number of pain sites was positively associated with age (RR 1.05, 95% CI 1.01–1.08), low household income (1.05, 1.01–1.08), unemployment (1.13, 1.06–1.19), any occupational exposure (1.17, 1.12–1.22), regular smoking (1.06, 1.02–1.11), and psychiatric symptoms (1.21, 1.17–1.26). Low education, living alone, physical inactivity, regular drinking or overweight were not significantly associated with the number of pain sites among men. Among women, the number of pain sites was positively associated with age (1.06, 1.04–1.10), unemployment (1.10, 1.05–1.15), any occupational exposure (1.10, 1.06–1.13), regular smoking (1.06, 1.02–1.10), overweight (1.08, 1.05–1.11), and psychiatric symptoms (1.19, 1.15–1.22). Living alone was negatively associated with the number of pain sites (0.95, 0.91–0.99). Low education, low household income, physical inactivity, and regular drinking were not significantly associated with the number of pain sites in women.

**Table 2:** The association of socioeconomic, occupational, psychological and lifestyle factors with number of pain sites.

	**Men (*n =* 3,615 observations)**								**Women (*n =* 3,842 observations)**						
	**Univariate models**				**Multivariable model**				**Univariate models**				**Multivariable model**		
	**RR**	**95% CI**	***P*-value**		**RR**	**95% CI**	***P*-value**		**RR**	**95% CI**	***P*-value**		**RR**	**95% CI**	***P*-value**
Age^a^	**1.03**	**1.00; 1.06**	**0.039**		**1.05**	**1.01; 1.08**	**0.005**		**1.07**	**1.04; 1.10**	**<** **0.001**		**1.06**	**1.04; 1.10**	**<** **0.001**
Low education	1.03	0.99; 1.07	0.211		1.01	0.97; 1.05	0.638		1.01	0.97; 1.04	0.736		1.00	0.96; 1.03	0.770
Living alone	1.01	0.97; 1.05	0.708		1.00	0.95; 1.04	0.824		**0.96**	**0.92; 0.99**	**0.036**		**0.95**	**0.91; 0.99**	**0.014**
Low household income	**1.08**	**1.05; 1.12**	**<** **0.001**		**1.05**	**1.01; 1.08**	**0.012**		**1.05**	**1.02; 1.08**	**0.002**		1.02	0.99; 1.05	0.297
Unemployment	**1.06**	**1.01; 1.12**	**0.013**		**1.13**	**1.06; 1.19**	**<** **0.001**		**1.05**	**1.01; 1.09**	**0.013**		**1.10**	**1.05; 1.15**	**<** **0.001**
Any occupational exposure	**1.12**	**1.09; 1.16**	**<** **0.001**		**1.17**	**1.12; 1.22**	**<** **0.001**		**1.05**	**1.02; 1.08**	**<** **0.001**		**1.10**	**1.06; 1.13**	**<** **0.001**
Physical inactivity	**1.06**	**1.03; 1.10**	**<** **0.001**		1.02	0.99; 1.06	0.229		**1.04**	**1.01; 1.07**	**0.021**		1.02	0.99; 1.05	0.275
Regular smoking	**1.10**	**1.06; 1.14**	**<** **0.001**		**1.06**	**1.02; 1.11**	**0.001**		**1.08**	**1.04; 1.11**	**<** **0.001**		**1.06**	**1.02; 1.10**	**0.002**
Regular drinking	1.01	0.98; 1.05	0.499		1.01	0.97; 1.05	0.666		**1.06**	**1.02; 1.10**	**0.004**		1.03	0.99; 1.07	0.094
Overweight	**1.04**	**1.01; 1.08**	**0.020**		1.03	0.99; 1.07	0.080		**1.10**	**1.07; 1.14**	**<** **0.001**		**1.08**	**1.05; 1.11**	**<** **0.001**
Psychiatric symptoms	**1.24**	**1.19; 1.29**	**<** **0.001**		**1.21**	**1.17; 1.26**	**<** **0.001**		**1.20**	**1.16; 1.23**	**<** **0.001**		**1.19**	**1.15; 1.22**	**<** **0.001**

*Rate ratios (RRs) for number of pain sites from generalized estimating equation (GEE) models. Bold denotes statistical significance*.

*CI, Wald's confidence interval; RR, Rate ratio*.

a *Time points compared with each other: age 46 vs. age 31*.

The independent effects of each occupational exposure are specified in Table 3. In the multivariable model of men, hard physical labor (1.07, 1.02–1.13), leaning forward (1.07, 1.02–1.12), back twisting (1.09, 1.04–1.15) and lifting loads of ≥ 1 kg (1.06, 1.01–1.12) were all independently associated with the number of pain sites. Among women, hard physical labor (1.07, 1.03–1.12), leaning forward (1.05, 1.02–1.10) and back twisting (1.06, 1.02–1.11) were associated with the number of pain sites.

**Table 3:** The specific associations between occupational physical exposures and number of pain sites.

	**Men (*n =* 3,615 observations)**								**Women (*n =* 3,842 observations)**						
	**Univariate models**				**Multivariable model^a^**				**Univariate models**				**Multivariable model^a^**		
	**RR**	**95% CI**	***P*-value**		**RR**	**95% CI**	***P*-value**		**RR**	**95% CI**	***P*-value**		**RR**	**95% CI**	***P*-value**
Hard physical labor	**1.19**	**1.15; 1.24**	**<** **0.001**		**1.07**	**1.02; 1.13**	**0.012**		**1.13**	**1.09; 1.17**	**<** **0.001**		**1.07**	**1.03; 1.12**	**0.002**
Leaning forward	**1.18**	**1.14; 1.22**	**<** **0.001**		**1.07**	**1.02; 1.12**	**0.004**		**1.09**	**1.06; 1.12**	**<** **0.001**		**1.05**	**1.02; 1.10**	**0.006**
Back twisting	**1.21**	**1.17; 1.26**	**<** **0.001**		**1.09**	**1.04; 1.15**	**<** **0.001**		**1.11**	**1.08; 1.15**	**<** **0.001**		**1.06**	**1.02; 1.11**	**0.005**
Constant moving	**1.10**	**1.06; 1.14**	**<** **0.001**		1.01	0.97; 1.06	0.557		1.02	0.99; 1.05	0.104		0.98	0.94; 1.01	0.216
Lifting loads of ≥ 1 kg	**1.16**	**1.12; 1.20**	**<** **0.001**		**1.06**	**1.01; 1.12**	**0.028**		**1.08**	**1.05; 1.11**	**<** **0.001**		1.03	0.99; 1.08	0.122

*Rate ratios (RRs) for number of pain sites from generalized estimating equation (GEE) models. Bold denotes statistical significance*.

*CI, Wald's confidence interval; RR, Rate ratio*.

a *Also adjusted for age, low education, living alone, low household income, unemployment, physical inactivity, smoking, drinking, overweight, and psychiatric symptoms*.

## Discussion

Among this general working-age population of Northern Finnish origin, multisite pain was independently associated with age, living alone, low household income, unemployment, occupational physical exposures, regular smoking, overweight, and psychiatric symptoms. The results of this study deepen the understanding of the underlying factors of and comorbidities behind multisite pain as well as help the development of pain relief strategies for working-age individuals with multisite pain.

The presence of psychiatric symptoms (i.e., anxiety and depression according to HSCL-25) was the strongest predictor of multisite pain among both sexes (RR 1.21 among men and 1.19 among women). It is evident that psychological and psychiatric factors such as depression, anxiety, catastrophic thinking, and fear of movement play an important role in the transition to chronic pain and disability (32). The present results provide further evidence of psychological and psychiatric comorbidity in multisite pain (5, 8–13, 33–35).

Our data underline the important role of socioeconomic and occupational determinants in multisite pain. Among both men and women, unemployment was independently associated with the number of pain sites (adjusted RR 1.13 among men and 1.10 among women), while household income had an independent association with the number of pain sites only among men. Our multivariable analysis thus provides further evidence of the independent negative role of unemployment in multisite pain (5). Engagement in physically strenuous tasks at work was associated with multisite pain. The presence of at least one occupational exposure (i.e., hard physical labor, leaning forward, back twisting, constant moving, lifting loads) showed a relatively high effect size among both sexes (RR 1.17 among men and 1.10 among women), outpowered by only psychiatric symptoms among both sexes and unemployment among women only. Further analysis of specific exposures identified hard physical labor, leaning forward and back twisting among both sexes and lifting loads among men as particularly relevant physical exposures in multisite pain. These exposures are likely to affect several anatomical areas and thus increase the number of potential pain sites. Occupational exposures have also been associated with multisite pain in previous studies (7, 15, 16). Surprisingly, in contrast to occupational physical features, leisure-time physical (in)activity was not associated with multisite pain among either sex.

Previous studies have associated multisite pain with smoking, physical inactivity and obesity (5, 6, 11, 16). The present multivariable analysis also included several health-related lifestyle factors which were studied for associations with multisite pain. The effect sizes were generally lower than those of the socioeconomic, occupational and psychological variables. Smoking was unsurprisingly associated with an increased risk of multisite pain among both sexes. Physical inactivity was not associated with multisite pain in the full multivariable models but did show univariate associations with pain in the preliminary models, suggesting that its independent role as a predictor of number of pain sites was outpowered by the remaining components. Overweight was associated with the number of pain sites among only women. Finally, women living alone were at a slightly lower risk of multisite pain.

The biopsychosocial perspective to medicine, as expedited by Engel (36), has been particularly influential in the field of chronic pain (2). Multidisciplinary biopsychosocial interventions including cognitive behavioral or functional items have shown stronger positive effects on pain and related disability than usual care provided by, e.g., general practitioners or physiotherapists alone (32, 37). The present findings clearly highlight the multifaceted nature of the socioeconomic, occupational, psychological and lifestyle factors underlying multisite pain, and thus underline the importance of biopsychosocial approaches to pain treatment and rehabilitation.

The main strength of this study was that it was based on a large general population birth cohort covering all sectors of the economy and occupations. Since all the participants were of the same age, any bias originating from macroeconomic fluctuations were avoided. The two-timepoint setting with data from the ages of 31 and 46 covers the phase of the life course during which one is expected to be fit for gainful work; from this perspective, using our results to further develop care and rehabilitation among individuals with multisite pain has notable economic potential. The study population is well-known, and we had access to a wide range of variables across their adulthood. As such, we were able to aggregate a wide variety of biopsychosocial variables accompanied by detailed variables of economic situation and various occupational physical loads in a set of models, in which the effects of their mutual correlations on the results could be accounted for and the independent effects of the variables could be identified. We ran separate models for men and women in order to study the predictors of multisite pain among each sex separately.

This study also had limitations. The questions regarding pain were in the dichotomous format of yes/no. Unfortunately, we did not have data on the intensity, frequency, bothersomeness, disability, or other dimensions of pain from the full course of the follow-up, and we were unable to supplement our dataset with these missing details in a retrospective manner. We also modeled the explanatory factors using dichotomous classifications in order to allow direct comparisons of effect size in terms of RRs. Finally, we had no data on pain or explanatory factors from the interval between the ages of 31 and 46. Except for BMI, all variables were self-reported, which may increase the risk of response bias in our data.

## Conclusions

In conclusion, we identified several independent predictors of multisite pain among working-age Finns of Northern origin. Among men, these were age, low household income, unemployment, occupational exposures, regular smoking, and psychiatric symptoms. Among women, they were age, living alone, unemployment, occupational exposures, regular smoking, overweight and psychiatric symptoms. It remains to be seen whether the same set of predictors can be identified in younger or older populations and whether the predictors are age dependent. Experimental studies are needed to improve and test interventions targeted at the main predictors identified by the present study to decrease the prevalence of potentially disabling multisite pain among working-age individuals.

## Data Availability Statement

The datasets presented in this article are not readily available because The Northern Finland Birth Cohort 1966 data is available from the University of Oulu, Infrastructure for Population Studies. Permission to use the data can be applied for research purposes *via* electronic material request portal (https://www.oulu.fi/nfbc/materialrequest). In the use of data, we follow the EU general data protection regulation (679/2016) and Finnish Data Protection Act. The use of personal data is based on cohort participant's written informed consent at his/her latest follow-up study, which may cause limitations to its use. Please, contact NFBC project center (NFBCprojectcenter@oulu.fi) and visit the cohort website (https://www.oulu.fi/nfbc) for more information. Requests to access the datasets should be directed to University of Oulu, Infrastructure for Population Studies, NFBCprojectcenter@oulu.fi.

## Ethics Statement

The studies involving human participants were reviewed and approved by Ethical Committee of the Northern Ostrobothnia Hospital District. The patients/participants provided their written informed consent to participate in this study.

## Author Contributions

V-HA, VR, LA-M, JK, and PO were involved in data collection. V-HA and PO analyzed the data. V-HA wrote the manuscript draft and prepared the figures. All authors read and approved the final manuscript.

## Conflict of Interest

The authors declare that the research was conducted in the absence of any commercial or financial relationships that could be construed as a potential conflict of interest.

## Publisher's Note

All claims expressed in this article are solely those of the authors and do not necessarily represent those of their affiliated organizations, or those of the publisher, the editors and the reviewers. Any product that may be evaluated in this article, or claim that may be made by its manufacturer, is not guaranteed or endorsed by the publisher.

## Abbreviations

BMI: Body mass index
CI: Confidence interval
GEE: Generalized estimating equations
HSCL-25: Hopkins Symptom Checklist-25
NFBC1966: Northern Finland Birth Cohort 1966
RR: Rate ratio.

## References

[1] International Association for the Study of Pain. IASP Terminology. (2020). Available online at: https://www.iasp-pain.org/terminology?navItemNumber=576 (accessed August 12, 2020).

[2] Gatchel R Peng Y Peters M Fuchs P Turk D. The biopsychosocial approach to chronic pain: scientific advances and future directions. Psychol Bull. (2007) 133:581–624. 10.1037/0033-2909.133.4.581 17592957

[3] Turk D Wilson H Cahana A. Treatment of chronic non-cancer pain. Lancet. (2011) 377:2226–35. 10.1016/S0140-6736(11)60402-9 21704872

[4] Cho N Kim I Lim S Kim H. Prevalence of widespread pain and its influence on quality of life: population study in Korea. J Korean Med Sci. (2012) 27:16–21. 10.3346/jkms.2012.27.1.16 22219608 PMC3247768

[5] Kamaleri Y Natvig B Ihlebaek C Benth J Bruusgaard D. Number of pain sites is associated with demographic, lifestyle, and health-related factors in the general population. Eur J Pain. (2008) 12:742–8. 10.1016/j.ejpain.2007.11.005 18160318

[6] Kindler L Jones K Perrin N Bennett R. Risk factors predicting the development of widespread pain from chronic back or neck pain. J Pain. (2010) 11:1320–8. 10.1016/j.jpain.2010.03.007 20488762 PMC2950865

[7] Toda K. The prevalence of fibromyalgia in Japanese workers. Scand J Rheumatol. (2007) 36:140–4. 10.1080/03009740600907949 17476621

[8] Croft P Rigby A Boswell R Schollum J Silman A. The prevalence of chronic widespread pain in the general population. J Rheumatol. (1993) 20:710–3. 8496870

[9] Gupta A Silman A Ray D Morriss R Dickens C MacFarlane G. The role of psychosocial factors in predicting the onset of chronic widespread pain: results from a prospective population-based study. Rheumatology (Oxford). (2007) 46:666–71. 10.1093/rheumatology/kel363 17085772

[10] Hunt I Silman A Benjamin S McBeth G Macfarlane G. The prevalence and associated features of chronic widespread pain in the community using the 'Manchester' definition of chronic widespread pain. Rheumatology (Oxford). (1999) 38:275–9. 10.1093/rheumatology/38.3.275 10325667

[11] Kamaleri Y Natvig B Ihlebaek C Bruusgaard D. Localized or widespread musculoskeletal pain: does it matter? Pain. (2008) 138:41–6. 10.1016/j.pain.2007.11.002 18077092

[12] MacFarlane G Croft P Schollum J Silman A. Widespread pain: is an improved classification possible? J Rheumatol. (1996) 23:1628–32. 8877936

[13] Macfarlane G Pye S Finn J Wu F Silman A Bartfai G. Investigating the determinants of international differences in the prevalence of chronic widespread pain: evidence from the European Male Ageing Society. Ann Rheum Dis. (2009) 68:690–5. 10.1136/ard.2008.089417 18653627

[14] McBeth J Macfarlane G Benjamin S Silman A. Features of somatization predict the onset of chronic widespread pain. Arthritis Rheum. (2001) 44:940–6. 10.1002/1529-0131(200104)44:4<940::AID-ANR151>3.0.CO;2-S 11315933

[15] Solidaki E Chatzi L Bitsios P Coggon D Palmer K Kogevinas M. Risk factors for new onset and persistence of multi-site musculoskeletal pain in a longitudinal study of workers in Crete. Occup Environ Med. (2013) 70:29–34. 10.1136/oemed-2012-100689 22864252 PMC3526653

[16] Solidaki E Chatzi L Bitsios P Markatzi I Plana E Castro F. Work related and psychological determinants of multisite musculoskeletal pain. Scand J Work Environ Health. (2010) 36:54–61. 10.5271/sjweh.2884 20011982 PMC3242043

[17] University of Oulu. Northern Finland Birth Cohort 1966. (2020). Available online at: http://urn.fi/urn:nbn:fi:att:bc1e5408-980e-4a62-b899-43bec3755243 (accessed August 12, 2020).

[18] Risku M. A historical insight on Finnish education policy from 1944 to 2011. Ital J Sociol Educ. (2014) 6:36–68. 10.14658/pupj-ijse-2014-2-3

[19] United Nations Economic Commission for Europe. Canberra Group Handbook on Household Income Statistics, 2nd edition. New York, NY; Geneva (2011).

[20] Oura P Rissanen I Junno J Harju T Paananen M. Lifelong smoking trajectories of Northern Finns are characterized by sociodemographic and lifestyle differences in a 46-year follow-up. Sci Sep. (2020) 10:16365. 10.1038/s41598-020-73334-3 PMC7529914 33004859

[21] Oura P Paananen M Niinimaki J Auvinen J Ala-Mursula L Junno J. Effect of occupational physical activities on vertebral dimensions in midlife in the Northern Finland Birth Cohort 1966. Occup Environ Med. (2016) 74:351–6. 10.1136/oemed-2016-104025 27864433

[22] Husten C. How should we define light or intermittent smoking? Does it matter? Nicotine Tob Res. (2009) 11:111–21. 10.1093/ntr/ntp010 19246425 PMC2658911

[23] Dawson D. Methodological issues in measuring alcohol use. Alcohol Res Health. (2003) 27:18–29. 15301397 PMC6676704

[24] World Health Organization. Body Mass Index - BMI. (2020). Available online at: http://www.euro.who.int/en/health-topics/disease-prevention/nutrition/a-healthy-lifestyle/body-mass-index-bmi (accessed August 12, 2020).

[25] Winokur A Winokur D Rickels K Cox D. Symptoms of emotional distress in a family planning service: stability over a four-week period. Br J Psychiatry. (1984) 144:395–9. 10.1192/bjp.144.4.395 6722401

[26] Nabbe P Le Reste J Guillou-Landreat M Munoz Perez M Argyriadou S Claveria A. Which DSM validated tools for diagnosing depression are usable in primary care research? A systematic literature review. Eur Psychiatry. (2017) 39:99–105. 10.1016/j.eurpsy.2016.08.004 27992813

[27] Veijola J Jokelainen J Laksy K Kantojarvi L Kokkonen P Jarvelin M. The Hopkins symptom checklist-25 in screening DSM-III-R axis-I disorders. Nord J Psychiatry. (2003) 57:119–23. 10.1080/08039480310000941 12745774

[28] Nettelbladt P Hansson L Stefansson C Borgquist L Nordstrom G. Test characteristics of the Hopkins symptom check list-25 (HSCL-25) in Sweden, using the Present State Examination (PSE-9) as a caseness criterion. Soc Psychiatry Psychiatr Epidemiol. (1993) 28:130–3. 10.1007/BF00801743 8378808

[29] Paananen M. Multi-site Musculoskeletal Pain in Adolescence: Occurrence, Determinants, and Consequences. Tampere: Acta Universitatis Ouluensis D 1133 (2011) 17:82.

[30] Twisk J. Applied Longitudinal Data Analysis for Epidemiology. Cambridge: Cambridge University Press (2003).

[31] Zeger S Liang K. Longitudinal data analysis for discrete and continuous outcomes. Biometrics. (1986) 42:121–30. 10.2307/2531248 3719049

[32] Guerrero A Maujean A Campbell L Sterling M. A systematic review and meta-analysis of the effectiveness of psychological interventions delivered by physiotherapists on pain, disability and psychological outcomes in musculoskeletal pain conditions. Clin J Pain. (2018) 34:838–57. 10.1097/AJP.0000000000000601 29554030

[33] Hartvigsen J Hancock M Kongsted A Louw Q Ferreira M Genevay S. What low back pain is and why we need to pay attention. Lancet. (2018) 391:2356–67. 10.1016/S0140-6736(18)30480-X 29573870

[34] Davies K Silman A Macfarlane G Nicholl B Dickens C Morriss R. The association between neighbourhood socio-economic status and the onset of chronic widespread pain: results from the EPIFUND study. Eur J Pain. (2009) 13:635–40. 10.1016/j.ejpain.2008.07.003 18782674 PMC2701988

[35] McBeth J Harkness E Silman A Macfarlane G. The role of the workplace low-level mechanical trauma, posture and environment in the onset of chronic widespread pain. Rheumatology. (2003) 42:1486–96. 10.1093/rheumatology/keg399 12867586

[36] Engel G. The need for a new medical model: a challenge for biomedicine. Science. (1977) 196:129–36. 10.1126/science.847460 847460

[37] van Erp R Huijnen I Jakobs M Kleijnen J Smeets R. Effectiveness of primary care interventions using a biopsychosocial approach in chronic low back pain: a systematic review. Pain Pract. (2019) 19:224–41. 10.1111/papr.12735 30290052 PMC7379915

